# Preliminary Assessment on Pathogenicity of an African Horse Sickness Virus Serotype 1 Strain in Guinea Pigs and Horses

**DOI:** 10.3390/microorganisms14071557

**Published:** 2026-07-16

**Authors:** Min Zhang, Xue-Feng Wang, Bo-Fan Fu, Si-Fan Guo, Lei Wang, Bing Zhang, Fei-Hu Guan, Ya-Fen Song, Xiao-Yue Yang, Ling-Ling Wang, Qian-Yi Zhang, Cheng-Huai Yang

**Affiliations:** 1 China Institute of Veterinary Drug Control, Beijing 102629, China; zmbooksea@sina.com (M.Z.);; 2 State Key Laboratory of Animal Disease Control and Prevention, Harbin Veterinary Research Institute, The Chinese Academy of Agricultural Sciences, Harbin 150069, China; wangxuefeng@caas.cn

**Keywords:** African horse sickness virus, pathogenicity, neutralizing antibody

## Abstract

African horse sickness (AHS), a noncontagious insect-borne disease affecting equids, is caused by the African horse sickness virus (AHSV) and is associated with extremely high morbidity and mortality. AHSV has nine distinct serotypes (AHSV-1 to AHSV-9). In a previous study, we characterized the genome and in vitro growth properties of AHSV/C, a cell-adapted derivative of an AHSV-1 strain imported into China 60 years ago, and verified its safety in mice. This study preliminarily explored the pathogenicity of AHSV/C in guinea pigs and horses (its natural host). Six groups of guinea pigs were inoculated with three doses of AHSV/C (10^7.5^, 10^6.5^, and 10^5.5^ TCID_50_ per animal) via both intraperitoneal and subcutaneous routes; two horses were subcutaneously inoculated with 10^5.5^ TCID_50_ of AHSV/C. All animals received a booster inoculation three weeks later. During the entire observation period, we mainly monitored clinical signs, body temperature, viremia, virus shedding and pathological changes (examined only in guinea pigs), and collected serum samples for neutralizing antibody detection. The results showed that no abnormal clinical signs, evident viremia, or virus shedding were observed in either species. Histopathological examinations revealed no evident lesions in guinea pigs. Neutralization tests showed that AHSV/C induced only low neutralizing antibody titers after primary inoculation, with a marked increase after booster administration. Collectively, this study preliminarily characterized the pathogenicity of AHSV/C in guinea pigs and horses, confirming that the strain showed no evident pathogenicity in test animals. These findings suggest that AHSV/C may be an attenuated strain. Further studies with larger sample sizes are required to validate these findings and explore their potential applications.

## 1. Introduction

African horse sickness (AHS) is a noncontagious, arthropod-borne, infectious disease of equids caused by the African horse sickness virus (AHSV). This virus belongs to the Orbivirus genus within the Sedoreovirinae subfamily of the Reoviridae family [1]. AHS is a highly pathogenic disease with extremely high morbidity and mortality rates among equids. It causes devastating losses to the horse industry and poses a severe threat to regional biosecurity and is listed as a notifiable disease by the World Organization for Animal Health (WOAH) [2].

The disease is characterized primarily by a persistent fever, edema affecting the lungs, pleura and subcutaneous tissues, and widespread hemorrhages across multiple organ systems [3]. The primary susceptible animals are all equids. Horses are the most vulnerable among them, with a mortality rate of up to 90% in unvaccinated populations. Mules and donkeys exhibit moderate susceptibility, while zebras act as asymptomatic carriers and natural reservoirs for virus transmission [4]. AHS is endemic to sub-Saharan Africa, with sporadic outbreaks occurring in North Africa. It has also been reported in several non-African countries, including Spain, Portugal, Saudi Arabia, Pakistan, India, Thailand and Malaysia [5,6,7,8].

Currently, there are no therapeutic treatments available for AHS, and consequently, control of the disease relies on preventive vaccination. The first attempts to control AHS through vaccination date back to the last century, when live attenuated vaccines (LAVs) were first used, which even today provide strong humoral and cellular immunity. LAV against AHS, produced by Onderstepoort Biological Products (OBP, Pretoria, South Africa), has been used in South Africa and other African countries for many decades and has effectively ensured the survival of horses in those regions. Apart from traditional live attenuated vaccines, various novel vaccines, including recombinant and inactivated vaccines, have been widely developed as alternative candidates for AHS prevention. However, none of these new vaccines have been commercially available at present. Although live attenuated vaccines carry risks of virulence reversion and genetic reassortment with wild strains, no safer alternative vaccines have been launched yet. Hence, live attenuated vaccines remain the primary choice for AHS control [9,10,11].

AHSV has nine distinct serotypes (AHSV-1 to AHSV-9), and they generally show minimal serological cross-reactivity [12]. VP7 is the major group-specific antigen of the virus, while VP2 is the major serotype-specific antigen [2]. The virulence of the virus varies among different serotypes: AHSV-1 to AHSV-8 are generally considered highly pathogenic to horses, resulting in high mortality rates. In contrast, AHSV-9 exhibits relatively lower pathogenicity yet is still capable of inducing severe clinical symptoms in horses. Historically, AHSV-4 caused outbreaks in Spain and Portugal between 1987 and 1990 [5]. In 2020, an outbreak of AHS induced by AHSV-1 occurred in Thailand, with a fatality rate as high as 93% [13,14]. This not only had a severe impact on local animal husbandry, but also posed a significant introduction risk to neighboring countries. As an AHS-free country, China is potentially at risk of AHS introduction and spread [15]. To strengthen the prevention and control measures, multiple research institutions in China have proactively launched relevant studies to focus on developing AHS diagnostic methods and candidate vaccines, as well as investigating the biological characteristics and distribution of its vector, *Culicoides* spp. [1,16,17,18,19].

Given the high pathogenicity of AHSV-1 and the escalating introduction risk to China from neighboring epidemic regions, developing serotype-specific candidate vaccines against AHSV-1 is of critical significance for targeted biosecurity stockpiling in China’s AHS prevention and control system. We therefore selected the AHSV/C cell-adapted strain as the research subject, which was derived from mouse brain-passaged AHSV-1 imported into China 60 years ago. Previously, we characterized the genome and in vitro growth properties of AHSV/C and verified its safety in mice [1]. On the basis of these findings, the present work preliminarily explores the pathogenicity of AHSV/C in guinea pigs and horses (the natural host of AHSV).

## 2. Materials and Methods

### 2.1. Virus Strain and Cell Culture

AHSV/C, an AHSV Cell-Adapted strain, is stored at the National Center for Veterinary Culture Collection (Beijing, China). It was derived from the AHSV mouse brain tissue strain, which was imported from abroad in the 1960s. This original strain was passaged three times in mouse brains, then adapted to African green monkey kidney cells (Vero cells) for 12 passages, followed by six passages of plaque purification. Relevant tests were completed as follows: Virus titer determination (10^7.5^ TCID_50_/mL); serotype identification (AHSV-1); whole-genome sequencing (sequence uploaded to NCBI: PX069092, PX069094, PX069096, PX069098, PX069100, PX069102, PX069104, PX069106, PX069108, PX069110); safety evaluation in mice [1].

The Vero CCL-81 cells, provided by the National Center for Veterinary Culture Collection, were cultured in Minimum Essential Medium (MEM; Gibco, Thermo Fisher Scientific, Waltham, MA, USA) at 37 °C with 5% CO_2_. The medium was supplemented with 8% heat-inactivated fetal bovine serum (FBS; Gibco, Thermo Fisher Scientific, Waltham, MA, USA).

### 2.2. Guinea Pigs

The animal study protocol was approved by the Institutional Animal Care and Use Committee (IACUC) of China Institute of Veterinary Drug Control (protocol code: IVDC (Fu) [2025] No. 00104; the date of approval is 27 March 2025). A total of 36 specific-pathogen-free (SPF) Hartley strain white short-haired guinea pigs weighing approximately 250 g, with equal numbers of males and females, were housed in an animal biosafety level 3 (ABSL-3) laboratory, where the environmental conditions were maintained at a temperature of 22 ± 2 °C and a relative humidity of 50 ± 5%. The guinea pigs were supplied with filtered air and maintained under a 12 h daily artificial light/dark cycle, with sterilized water and irradiated feed provided ad libitum. Standard Operating Procedures for laboratory animals were implemented throughout the study to minimize stress and pain in the experimental subjects. If the animals exhibited severe clinical symptoms at any stage of the experiment, euthanasia was performed in accordance with ethical guidelines.

### 2.3. Horses

The animal study protocol was approved by the Institutional Animal Care and Use Committee (IACUC) of China Institute of Veterinary Drug Control (protocol code: IVDC (Fu) [2023] No. 00210; the date of approval is 24 October 2023). Owing to the stringent biosafety protocols, limited facility capacity, and rigorous operational requirements of the ABSL-3 laboratory, only two DeBao ponies were selected for the experiment. The two horses, aged 2–3 years, were healthy, consisting of one female (H1) and one male (H2).

The horses were housed in an ABSL-3 laboratory, where the environmental conditions were maintained at a temperature of 20 ± 2 °C and a relative humidity of 50 ± 5%. A daily 12 h artificial light/dark cycle was implemented, and the horses were provided with free access to dry hay, concentrated feed, and sterilized water. The feeding area was cleaned daily to maintain good hygiene, and a sufficiently large space was allocated to ensure the horses had adequate room for movement. Standard Operating Procedures for laboratory animals were implemented throughout the study to minimize stress and pain in the experimental subjects. If the animals exhibited severe clinical symptoms at any stage of the experiment, euthanasia was performed in accordance with ethical guidelines.

Prior to the experiment, the two horses were screened for antigens and antibodies against common equine infectious diseases, and their basal body temperatures (rectal temperature) were monitored for 3 consecutive days. The results showed that both horses tested negative for the common pathogens, including AHSV, Equine Infectious Anemia Virus (EIAV), Equine Influenza Virus (EIV), Equine Herpesvirus (EHV), and Equine Arteritis Virus (EAV). For antibody detection, all virus antibody test results were negative except that the antibody test result for EHV-4 was positive. Additionally, their body temperatures measured over the 3 days were all within the normal range (37.5–38.5 °C). These findings indicate that the two horses are in good health and suitable for this experiment.

All personnel involved in animal handling, experimental operation and outcome evaluation possess valid and legal professional qualifications for laboratory animal practitioners, have completed special training on laboratory animal welfare and ethics, and strictly complied with the ethical approval requirements of this study throughout the entire operation process.

### 2.4. Real-Time RT-PCR

Referring to the real-time RT-PCR method recommended by the WOAH Manual of Diagnostic Tests and Vaccines for Terrestrial Animals (the WOAH Terrestrial Manual) [12], the conserved VP7 gene fragment of AHSV was amplified. The primer sequences were 5′-CCA-GTA-GGC-CAG-ATC-AAC-AG-3′ and 5′-CTA-ATG-AAA-GCG-GTG-ACC-GT-3′. The probe is 5′-FAM-GCT-AGC-AGC-CTA-CCA-CTA-MGB-3′. The reaction cycle for real-time one-step RT-PCR was as follows: 48 °C for 25 min; 95 °C for 10 min; then 40 cycles: 95 °C (15 s), 55 °C (35 s), 72 °C (30 s); and fluorescence data were acquired at the end of the 72 °C step. For each test batch, positive controls, negative controls and non-vaccinated animal controls were included. The assay was deemed positive if a typical amplification curve appeared with a cycle threshold (Ct)value ≤ 35 within 40 PCR cycles, inconclusive if a typical amplification curve was observed with 35 < Ct ≤ 40, and negative if only a flat amplification curve was obtained with no Ct value detected.

### 2.5. Neutralization Assay

The collected serum was serially diluted 2-fold with cell culture medium. AHSV/C virus was also diluted in cell culture medium to obtain a concentration of 100 TCID_50_/0.1 mL (TCID_50_ = 50% tissue culture infectious dose). A total of 1 mL of serum at each dilution was mixed with an equal volume of diluted virus solution for neutralization, and then incubated at 37 °C for 1 h. The solution was then inoculated into 5 wells of a 96-well plate containing Vero cells, with 0.1 mL solution per well. Meanwhile, 5 wells of positive control, 5 of negative control, 5 of virus control, and 5 of normal cell control were set up. In the positive control wells, diluted virus solution was mixed with an equal volume of AHSV-1-positive serum (provided by the Pribright Institute, an AHS reference laboratory of WOAH). In the negative wells, diluted virus solution was mixed with an equal volume of AHSV-1-negative serum. In the virus control, diluted virus solution was mixed with an equal volume of cell culture medium. In the normal cell control wells, Vero cells were inoculated with only the cell culture medium. The plates were incubated at 37 °C and 5% CO_2_ level for 120 h, and the number of wells showing Cytopathic Effect (CPE) in each group was observed. When the positive control and normal cell control show no CPE, while the negative control and virus control show CPE, it indicates that the test conditions are valid. Record the number of wells with no CPE (protection) for each serum dilution, and calculate the reciprocal 50% serum neutralizing antibody titer (SN_50_) using the Reed–Muench method.

### 2.6. Pathogenicity of AHSV/C in Guinea Pigs

To determine the pathogenicity of AHSV/C in guinea pigs, 36 guinea pigs (equal numbers of males and females) were allocated to 6 experimental groups (6 animals per group) via sex-stratified block randomization. According to the inoculation method recommended by WOAH for AHS safety evaluation [12], intraperitoneal injection was selected as the inoculation route, and subcutaneous injection was additionally included. The 6 experimental groups were established by the combination of three AHSV/C titers (10^7.5^, 10^6.5^, 10^5.5^ TCID_50_/mL) and two inoculation routes, with each titer-route pair corresponding to one independent group. Separately, 6 guinea pigs were randomly assigned to 2 control groups (3 animals per group) using simple randomization, receiving uninfected Vero cell culture via intraperitoneal or subcutaneous injection, respectively. Each guinea pig was administered 1 mL of inoculum. Subsequently, four key parameters were assessed to comprehensively evaluate the safety of AHSV/C in guinea pigs, with the specific procedures described as follows (Table 1):

- Clinical Symptoms Monitoring: The guinea pigs were examined daily for 14 consecutive days post-inoculation. Clinical manifestations, including mental status, food and water intake, mobility, and any abnormal signs, were recorded in detail.
- Viremia Detection: On 1, 4, 7, 10, and 14 days post-inoculation (dpi), cardiac blood collection was performed using a non-lethal blood sampling method. Specifically, each guinea pig was first gently restrained in a dedicated holder, and the skin over the left precordial region was disinfected with 75% medical alcohol. Next, a sterile disposable fine-gauge blood collection needle was inserted slowly to a shallow depth into the intercostal space between the 3rd and 4th ribs, approximately 0.5 cm lateral to the sternum, with the needle tip directed toward the cardiac apex, taking care to avoid tissue damage. Approximately 0.5 mL of blood was collected per animal immediately once blood flow was observed. After collection, the needle was withdrawn promptly, and the sampling site was pressed firmly with a sterile cotton ball for 30 s to achieve complete hemostasis. Finally, following anticoagulation treatment, viral nucleic acid in the blood samples was quantified via real-time RT-PCR, as detailed in Section 2.4.
- Virus Shedding Assessment: At the aforementioned dpi time points, oropharyngeal and anal swabs were collected from each guinea pig and immediately immersed in 1 mL of sterile physiological saline. After incubation at 2–8 °C for 2 h, the resulting suspensions were vortexed thoroughly and centrifuged at 3000× *g* for 10 min; the supernatants were then harvested, and viral RNA was detected using the real-time RT-PCR protocol described in Section 2.4.
- Pathological Analysis: On 14 dpi, two guinea pigs from each experimental group and one guinea pig from the control group were euthanized by CO_2_ overdose. Necropsy was then performed immediately, and gross pathological changes in all major tissues and organs were recorded systematically. The pathological sections of the heart, liver, spleen, lung, and kidney of guinea pigs were prepared at Wuhan Sevier Biotechnology Co., Ltd. (Wuhan, China) for histopathological examination.

### 2.7. Pathogenicity of AHSV/C in Horses

The AHSV/C was diluted to contain 10^5.5^ TCID_50_ per 5 mL. According to the inoculation method recommended by WOAH for AHS safety evaluation [12], two horses (H1 and H2) were subcutaneously injected with 5 mL of the diluted AHSV/C suspension in the neck. Subsequently, four key parameters were assessed to comprehensively evaluate the safety of AHSV/C in horses, with the specific procedures described as follows (Table 2):

- Clinical Symptoms Monitoring: The horses were examined daily for 14 consecutive days post-inoculation. Clinical manifestations, including mental status, food and water intake, mobility, and any abnormal signs, were recorded in detail.
- Rectal Temperature Measurement: The rectal temperatures measured at a fixed time point 3 days pre-inoculation were used as the basal body temperatures. The same fixed time point was adopted to measure the horses’ rectal temperature every day for 14 consecutive days post inoculation, with the data recorded systematically.
- Viremia Detection: On 0, 1, 3, 5, 7, 9, 11 and 13 dpi (with samples at 0 dpi serving as pre-inoculation negative controls), approximately 1 mL of blood was collected from the jugular vein of each of the two horses. Following anticoagulation, viral nucleic acid in the blood was detected via the real-time RT-PCR method described in Section 2.4.
- Virus Shedding Assessment: On 0, 1, 3, 5, 7, 9, 11 and 13 dpi (with samples at 0 dpi serving as pre-inoculation negative controls), nasal swabs and anal swabs were collected from each horse and placed in 1 mL of physiological saline. After incubation at 2–8 °C for 2 h, the suspensions were vortexed thoroughly and centrifuged at 3000× *g* for 10 min; the supernatants were then harvested, and the viral RNA was detected using the real-time RT-PCR method as described in Section 2.4.

### 2.8. Detection of Neutralizing Antibodies

Serum samples were collected from guinea pigs and horses for neutralizing antibody detection during the study. All animals received a booster inoculation 21 days after the first inoculation.

For guinea pigs, cardiac blood samples (approximately 0.5 mL/animal) were collected on days 7, 14 and 21 post primary inoculation, and on days 7, 14, 21, 28 and 35 post booster inoculation (corresponding to days 28, 35, 42, 49 and 56 post primary inoculation). For horses, blood samples (approximately 5 mL/animal) were collected from the jugular vein on days 0, 7, 14 and 21 post primary inoculation (pre-inoculation serum at day 0 served as negative control), and on days 7 and 14 post booster inoculation (corresponding to days 28 and 35 post primary inoculation). All sera were separated and stored at −20 °C until use.

Neutralizing antibody titers were determined as described in Section 2.5, with three technical replicates per sample. The SN_50_ titer, defined as the reciprocal of the highest serum dilution that inhibited 50% of CPE, was calculated using the Reed–Muench method. Results were expressed as the mean ± standard deviation of the original SN_50_ titers, and the dynamic changes in antibodies were plotted after converting the values to log_2_ scale.

## 3. Results

### 3.1. Assessment of Pathogenicity of AHSC/C in Guinea Pigs and Horses

No obvious pathogenic manifestations were detected in guinea pigs (across different doses and routes) and horses (at the tested dose) in this preliminary trial.

**Figure 1:**
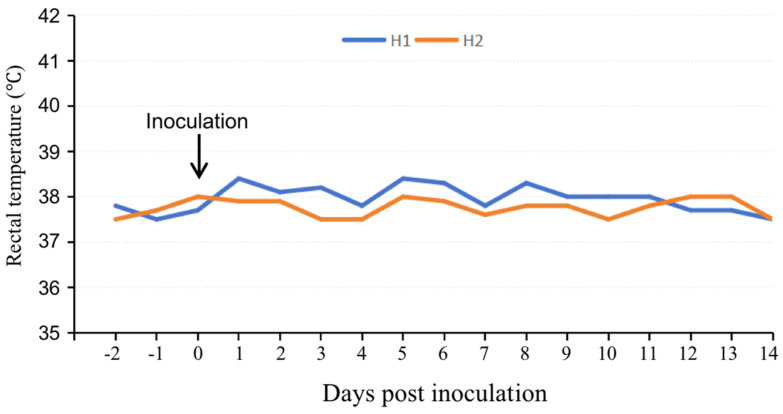
Rectal temperatures of two horses at the indicated time points before and after AHSV/C inoculation. Day 0 denotes the day of inoculation. The rectal temperatures of both horses remained within the normal range (37.5–38.5 °C) throughout the observation period. H1, horse 1; H2, horse 2.

Guinea pigs and horses inoculated with AHSV/C were monitored continuously for 14 days post-inoculation. Throughout the observation period, all animals maintained normal food intake, water consumption, mental state, and hair coat, with no clinical symptoms observed. Daily rectal temperature measurements of the two horses showed that their temperatures remained within the normal range (37.5–38.5 °C) and never exceeded 39 °C, the upper limit of equine rectal temperature for safety testing specified by WOAH. Specific temperature profiles are presented in Figure 1.

The presence of viremia and virus shedding in inoculated horses and guinea pigs was monitored using a real-time RT-PCR assay recommended by WOAH, with blood and swabs (nasal for horses, oropharyngeal for guinea pigs) sampled at predetermined time points post inoculation for nucleic acid testing. The assay cut-off value for positivity was Ct ≤ 35. All negative controls yielded negative results, and all positive controls in each PCR run showed Ct values ≤ 35, confirming valid amplification of the detection system. All samples from inoculated animals had Ct values above 35 (Table 3 and Table 4, Appendix A Table A1 and Table A2), consistent with the negative control group. Ct values of 37 to 40 were observed in several samples from both experimental and control groups, which were attributed to background interference and non-specific amplification. No biologically meaningful AHSV nucleic acid was detected in blood and swab samples of test animals in this study. Collectively, at the tested doses, AHSV/C did not induce evident viremia or virus shedding in horses and guinea pigs.

**Figure 2:**
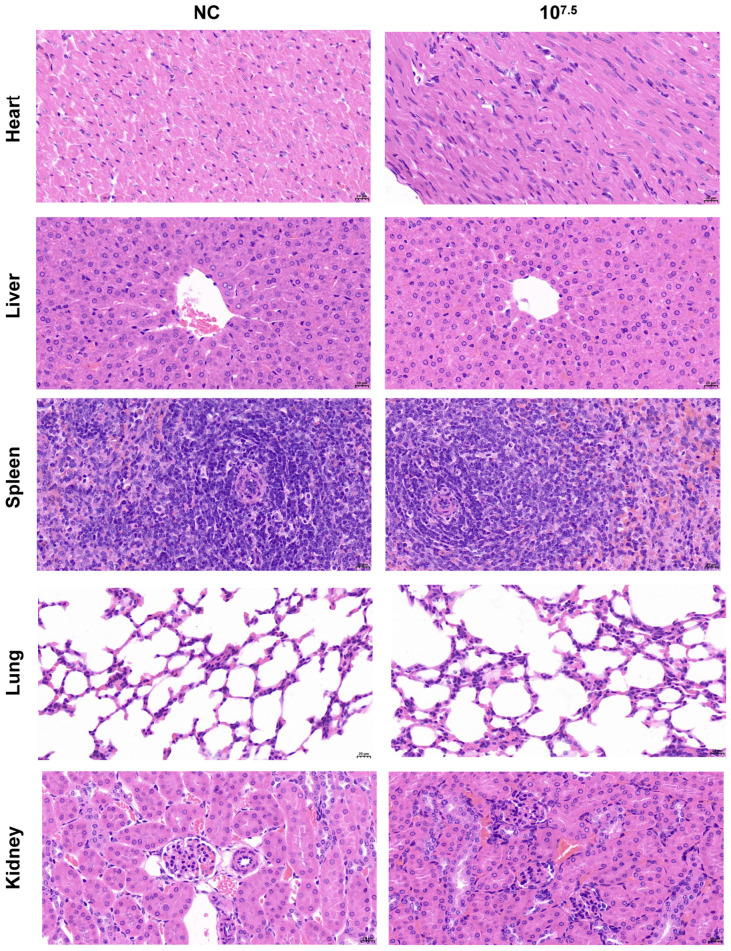
Microscopic features of the heart, liver, spleen, lung and kidney from guinea pigs intraperitoneally injected with AHSV/C at a dose of 10^7.5^ TCID_50_/mL, as revealed by hematoxylin-eosin (HE) staining. No obvious histopathological lesions were observed in all the examined tissues. NC, negative control. Magnification: 400×.

**Figure 3:**
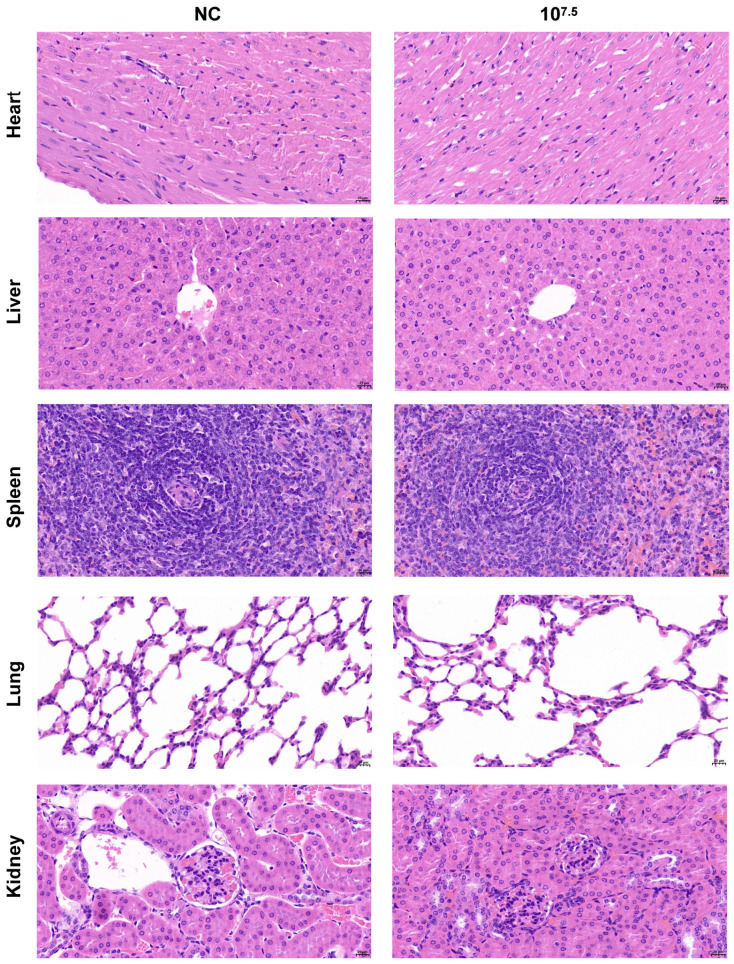
Microscopic features of the heart, liver, spleen, lung and kidney from guinea pigs subcutaneously injected with AHSV/C at a dose of 10^7.5^ TCID_50_/mL, as revealed by hematoxylin-eosin (HE) staining. No obvious histopathological lesions were observed in all the examined tissues. NC, negative control. Magnification: 400×.

For pathological assessment, two guinea pigs per inoculated group and one guinea pig from the control group were euthanized for complete necropsy. Gross pathological observation was conducted on key organs, including the heart, liver, spleen, lung, and kidney. No obvious macroscopic pathological alterations (e.g., congestion, hemorrhage, necrosis, atrophy, or abnormal texture changes) were observed in any of the examined organs across all groups. Subsequently, the heart, liver, spleen, lung and kidney tissues from each necropsied guinea pig were harvested, fixed, embedded, sectioned, and stained with hematoxylin and eosin (HE) for histopathological examination. Microscopic observation revealed that all the evaluated tissues from guinea pigs in the inoculated groups exhibited intact tissue architecture and clear cellular boundaries, with no detectable pathological lesions such as inflammatory cell infiltration, tissue degeneration, necrosis, or fibrosis. Representative histopathological images of the high-dose group (10^7.5^ TCID_50_/mL) are presented in Figure 2 and Figure 3, while the corresponding images of the medium-dose and low-dose groups are provided in the Supplementary Materials (Figures S1 and S2).

These findings are consistent with our previous safety evaluation results in mice [1], further supporting the low pathogenicity profile of AHSV/C.

### 3.2. Neutralizing Antibody Detection

**Figure 4:**
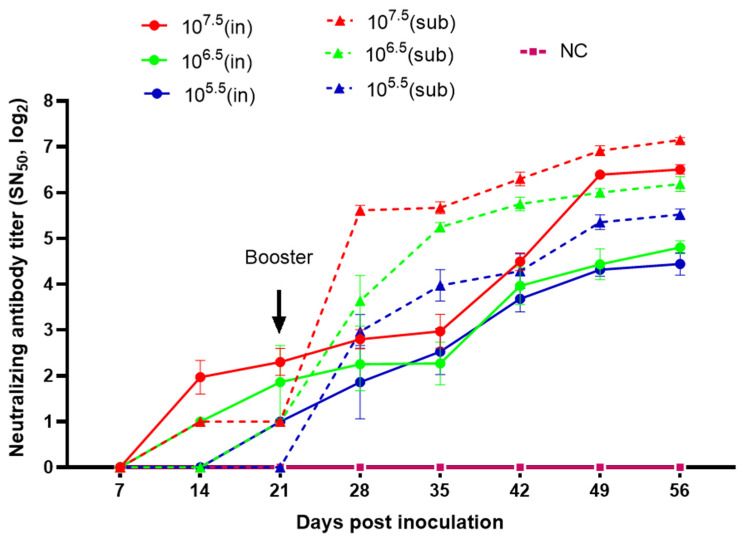
Dynamics changes in serum neutralizing antibody titers (SN_50_, log_2_) in guinea pigs inoculated with graded doses of AHSV/C (10^5.5^, 10^6.5^, 10^7.5^TCID_50_). SN_50_ was calculated by the Reed–Muench method with three technical replicates per sample, and log_2_-transformed SN_50_ values were plotted to represent neutralizing antibody level dynamics. Note: in, intraperitoneal injection; sub, subcutaneous injection; NC, negative control; SN_50_, the reciprocal 50% serum neutralizing antibody titer.

**Figure 5:**
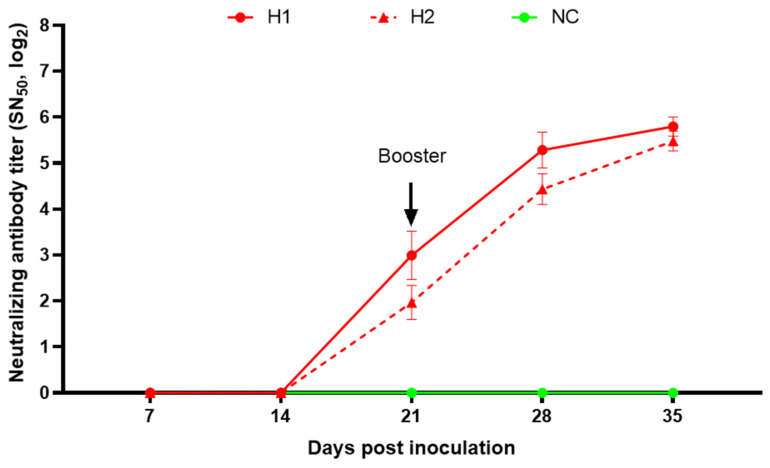
Dynamics changes in serum neutralizing antibodies (SN_50_, log_2_) in horses inoculated with graded doses of AHSV/C (10^5.5^, 10^6.5^, 10^7.5^TCID_50_). SN_50_ was calculated by the Reed–Muench method with three technical replicates per sample, and log_2_-transformed SN_50_ values were plotted to represent neutralizing antibody level dynamics. Note: H1, horse 1; H2, horse 2; NC, negative control; SN_50_, the reciprocal 50% serum neutralizing antibody titer.

Serum samples were collected from guinea pigs and horses at scheduled time points to detect neutralizing antibody levels following AHSV/C inoculation. Of note, sera from guinea pigs were pooled prior to testing; thus, individual variation could not be evaluated, and statistical analysis was not performed. Neutralizing antibody titers (SN_50_) are presented in Appendix A Table A3 and Table A4, and their dynamics (log_2_-transformed values) are shown in Figure 4 and Figure 5. A consistent upward trend in antibody levels was observed in both species after inoculation.

SN_50_ titers were low after the primary inoculation. In guinea pigs, titers (2 to 4) were first detected in the high- and medium-dose groups on day 14, while no titer was found in the low-dose group. By day 21, low-level SN_50_ titers (2 to 5) were detected in all three dose groups. In horses, only the tested dose was used, and low-level SN_50_ titers (4 to 8) became detectable starting on day 21.

All animals received a booster inoculation on day 21. Following the booster, SN_50_ titers showed a marked increase in all groups. In the high-dose subcutaneous group of guinea pigs, the SN_50_ titer reached 49 on day 7 post-booster. By day 21 post-booster, SN_50_ titers exceeded 20 in all three subcutaneous dose groups, whereas titers in the intraperitoneal groups remained lower across all doses. For horses, SN_50_ titers exceeded 20 in both animals by day 7 after the booster.

## 4. Discussion

In this study, we performed experiments in guinea pigs and horses to preliminarily evaluate the pathogenicity of AHSV/C. The results preliminarily demonstrate that the tested strain exhibits no evident pathogenicity to both animal species at the administered doses, and is capable of inducing specific neutralizing antibodies.

Guinea pigs were challenged with three viral doses via intraperitoneal and subcutaneous routes, while horses received a single dose by subcutaneous inoculation. In accordance with the WOAH Terrestrial Manual [12], we monitored clinical signs, blood viral loads and virus shedding over 14 consecutive days post AHSV/C inoculation. Daily body temperature measurement was performed on horses, and guinea pigs underwent gross necropsy and histopathological examination at the end of the observation period. Throughout the observation period, all inoculated animals maintained normal mental status and feeding activity, with no lethargy, emaciation or other abnormal clinical manifestations. The body temperature of horses did not exceed 39 °C, the critical temperature for safety assessment of live African horse sickness vaccines stipulated by the WOAH Terrestrial Manual. Even at the highest viral dose, neither inoculation route induced pathogenic reactions in guinea pigs. After dissection, no gross lesions or abnormal histological changes were found in the visceral organs of guinea pigs. Combined with our previous findings in mice, these consistent results across multiple animal species further confirm that AHSV/C possesses stable low pathogenicity. According to the WOAH Terrestrial Manual, mice, guinea pigs and horses are standard experimental animals for the safety evaluation of live African horse sickness vaccines, which also makes our animal selection fully compliant with international evaluation standards. Due to animal ethics requirements and subsequent experimental arrangements, pathological examinations were not conducted on horses.

For pathogenic viruses, the ability to replicate efficiently in the host and induce persistent viremia is the core manifestation of virulence. Virulent AHSV can proliferate massively in vivo, causing severe tissue damage, typical clinical symptoms, persistent viremia and continuous virus shedding, which are the main driving factors for disease prevalence and horizontal transmission between animals. It has been reported that viremia typically persists for 4 to 8 days in horses infected with AHSV, with a maximum duration of up to 21 days [20]. In the Thailand AHS outbreak caused by AHSV-1, viral load in the blood of infected horses corresponded to Ct values of 16–26 [8]. In the present study, we used the qPCR assay established by Agüero et al. [21], which is the recommended diagnostic method for AHSV by the WOAH, to detect AHSV nucleic acid in peripheral blood, oropharyngeal (or nasal) swabs, and anal swabs from horses and guinea pigs. The qPCR test results showed that most samples yielded no Ct values, while only a small number of samples (including some from the control group) had Ct values ranging from 37 to 40. According to the WOAH criteria, these results were classified as inconclusive. Considering that similar Ct values were also detected in the control group, these signals were most likely below the lower limit of detection of the assay and presumed to be caused by background interference and non-specific amplification. This indicates that virtually no biologically meaningful AHSV nucleic acid was detected in all samples. Nevertheless, the presence of trace, transient viral nucleic acid or nucleic acid below the detection limit cannot be completely ruled out. No evident viremia or virus shedding was detected in AHSV/C-inoculated guinea pigs and horses, indicating that the strain did not establish systemic infection in these animals at the tested doses, with a low risk of horizontal transmission. Collectively, these preliminary data further indicate that the AHSV/C strain lacks pathogenicity in guinea pigs and horses.

To evaluate the humoral immune response induced by AHSV/C, we determined serum neutralizing antibody titers in inoculated guinea pigs and horses. Only low levels of neutralizing antibodies were detected after primary inoculation, whereas antibody levels increased markedly following booster immunization. Since this strain fails to induce clinical disease in experimental animals while efficiently eliciting neutralizing antibody responses, it has potential as a candidate strain for further research. Nevertheless, comprehensive follow-up studies are still needed to fully verify its immunogenic performance.

Although this preliminary work yields meaningful results, it has certain inherent limitations. First, only two horses were used in this experiment with a single inoculation dose and route. This constraint mainly arose from stringent biosafety protocols, limited laboratory capacity, and strict operational requirements of the ABSL-3 facility. Although strict homogenization was applied to the basic physiological indices, housing environment and experimental procedures of the two horses throughout the experiment to minimize potential bias from individual animal variation, the small sample size inevitably reduced the statistical power of the study. Accordingly, the findings of this work are only preliminary observations and cannot serve as definitive conclusions at the population level. Second, only short-term sampling and observation were conducted after inoculation, and long-term monitoring was not implemented, so the long-term safety of AHSV/C cannot be fully evaluated. Third, no virulent challenge test was performed. China is an African horse sickness-free country, and virulent strains are currently unavailable domestically, making it impossible to verify the immune protective effect of the strain.

In follow-up work, we will arrange multiple repeated experiments to expand the number of experimental horses and set multiple dose groups and inoculation routes so as to obtain more comprehensive data to analyze the biological characteristics of AHSV/C. We will also strengthen international academic exchanges and explore feasible technical schemes to complete relevant immunogenicity evaluation studies.

In conclusion, the results of this study demonstrate that AHSV/C exhibits no obvious pathogenicity to guinea pigs and horses under the current experimental conditions, suggesting that it is likely an avirulent strain. Although the strain can induce the production of neutralizing antibodies, its protective efficacy remains to be further verified. Additionally, this work has generated valuable reference materials (e.g., AHSV-1 antisera) for the development of AHSV-1 diagnostic reagents—resources that are particularly precious for China, an AHS-free country.

## Figures and Tables

**Table 1:** Experimental Time Nodes and Operation Details for AHSV/C pathogenicity in Guinea Pigs.

Detection Index	Sampling/Operation Time Point	Specific Operation Details
Clinical Symptom Monitoring	1–14 dpi (daily)	Daily examination of guinea pigs for clinical signs, including mental status, food/water intake and mobility, with detailed recording of any abnormal manifestations
Viremia Detection	1, 4, 7, 10, 14 dpi	Non-fatal cardiac blood collection: Approximately 0.5 mL blood was sampled from two experimental guinea pigs per group and one control guinea pig per group; viral nucleic acid detection via real-time RT-PCR after anticoagulation (see Section 2.4 for details)
Viral Shedding Assessment	1, 4, 7, 10, 14 dpi	Collection of oropharyngeal and anal swabs from each guinea pig; immersion in 1 mL sterile physiological saline; incubation at 2–8 °C for 2 h; viral RNA detection via real-time RT-PCR (see Section 2.4 for details)
Pathological Analysis	14 dpi	Humane euthanasia of two experimental guinea pigs per group and one control guinea pig by CO_2_ overdose; necropsy and systematic recording of gross pathological changes; preparation and light microscopic examination of heart, liver, spleen, lung and kidney sections for histopathological analysis

Note: dpi, days post-inoculation; RT-PCR, reverse transcription polymerase chain reaction.

**Table 2:** Experimental Time Nodes and Operation Details for AHSV/C pathogenicity in horses.

Detection Index	Sampling/Operation Time Point	Specific Operation Details
Clinical Symptom Monitoring	1–14 dpi (daily)	Daily examination of horses for clinical signs, including mental status, food/water intake and mobility, with detailed recording of any abnormal manifestations
Rectal Temperature Measurement	3 days pre-inoculation, 1–14 dpi (daily)	Measure rectal temperature of horses at a fixed time every day and document the data systematically
Viremia Detection	0, 1, 3, 5, 7, 9,11,13 dpi	Approximately 1 mL of blood was collected from the jugular vein of two horses; the viral nucleic acid in the blood was detected using the real-time RT-PCR method after anticoagulation (see Section 2.4 for details)
Viral Shedding Assessment	0, 1, 3, 5, 7, 9,11,13 dpi	Collection of nasal swabs and anal swabs from each horse; immersion in 1 mL sterile physiological saline; incubation at 2–8 °C for 2 h; viral RNA detection via real-time RT-PCR (see Section 2.4 for details)

Note: dpi, days post-inoculation; RT-PCR, reverse transcription polymerase chain reaction.

**Table 3:** Detection of viral RNA (Ct values) in the blood of guinea pigs inoculated with AHSV/C using the real-time RT-PCR method.

Dpi	GP	10^7.5^ TCID_50_		10^6.5^ TCID_50_		10^5.5^ TCID_50_		Control Group		Positive Control	Negative Control
		In	Sub	In	Sub	In	Sub	In	Sub		
1	GP1	39.15	No Ct	No Ct	No Ct	No Ct	39.43	39.21	No Ct	27	No Ct
	GP2	No Ct	No Ct	No Ct	39.01	No Ct	No Ct	N/A	N/A		
4	GP1	No Ct	37.53	37.07	No Ct	38.15	No Ct	No Ct	No Ct		
	GP2	No Ct	No Ct	No Ct	No Ct	No Ct	38.96	N/A	N/A		
7	GP1	38.69	No Ct	No Ct	38.68	No Ct	No Ct	38.03	No Ct		
	GP2	No Ct	No Ct	No Ct	No Ct	38.98	No Ct	N/A	N/A		
10	GP1	38.14	39.04	39.91	No Ct	No Ct	No Ct	No Ct	39.27		
	GP2	No Ct	No Ct	No Ct	No Ct	No Ct	39.02	N/A	N/A		
14	GP1	No Ct	39.94	No Ct	39.65	No Ct	No Ct	No Ct	No Ct		
	GP2	No Ct	No Ct	No Ct	No Ct	39.04	No Ct	N/A	N/A		

Note: Dpi, days post inoculation; GP, guinea pig; In, intraperitoneal injection; Sub, subcutaneous injection; N/A, not applicable.

**Table 4:** Detection of viral RNA (Ct values) in blood, nasal swabs and anal swabs from two horses inoculated with AHSV/C using the real-time RT-PCR method.

Dpi	Blood		Nasal Swabs		Anal Swabs	
	H1	H2	H1	H2	H1	H2
1	No Ct	No Ct	No Ct	38.63	No Ct	No Ct
3	39.16	No Ct	No Ct	No Ct	39.81	No Ct
5	No Ct	39.94	38.79	No Ct	No Ct	No Ct
7	No Ct	No Ct	No Ct	38.71	No Ct	39.96
9	No Ct	No Ct	No Ct	No Ct	No Ct	No Ct
11	39.02	No Ct	No Ct	No Ct	38.72	No Ct
13	No Ct	No Ct	No Ct	No Ct	No Ct	No Ct
Pre-inoculation negative control	No Ct	No Ct	39.94	No Ct	No Ct	39.18
Positive control	24.8					
Negative control	No Ct					

Note: Dpi, days post inoculation; H1, horse 1; H2, horse 2.

## Data Availability

The original contributions presented in this study are included in the article/Supplementary Materials. Further inquiries can be directed to the corresponding authors.

## Supplementary Materials

The following supporting information can be downloaded at: https://www.mdpi.com/article/10.3390/microorganisms14071557/s1, Figure S1: Microscopic features of the heart, liver, spleen, lung and kidney from guinea pigs intraperitoneally injected with AHSV/C at a dose of 10^6.5^ and 10^5.5^ TCID_50_/mL, as revealed by hematoxylin-eosin (HE) staining. No obvious histopathological lesions were observed in all the examined tissues. NC, negative control. Magnification: 400×; Figure S2: Microscopic features of the heart, liver, spleen, lung and kidney from guinea pigs subcutaneously injected with AHSV/C at a dose of 10^6.5^ and 10^5.5^ TCID_50_/mL, as revealed by hematoxylin-eosin (HE) staining. No obvious histopathological lesions were observed in all the examined tissues. NC, negative control. Magnification: 400×.

## Abbreviations

The following abbreviations are used in this manuscript:

AHSV	African horse sickness virus
AHS	African horse sickness
RT-PCR	Reverse transcription-polymerase chain reaction
TCID_50_	50% tissue culture infectious dose
EIAV	Equine Infectious Anemia Virus
EIV	Equine Influenza Virus
EHV	Equine Herpesvirus
SN_50_	The reciprocal 50% serum neutralizing antibody titer

## Appendix A

**Table A1:** Detection of viral RNA (Ct values) in oropharyngeal swabs from guinea pigs inoculated with AHSV/C using the real-time RT-PCR method.

Dpi	GP	10^7.5^ TCID_50_		10^6.5^ TCID_50_		10^5.5^ TCID_50_		Control Group		Positive Control	Negative Control
		In	Sub	In	Sub	In	Sub	In	Sub		
1	GP1	No Ct	No Ct	No Ct	No Ct	No Ct	39.96	No Ct	39.42	24.14	No Ct
	GP2	No Ct	No Ct	No Ct	No Ct	No Ct	No Ct	No Ct	No Ct		
	GP3	No Ct	No Ct	No Ct	No Ct	No Ct	No Ct	No Ct	No Ct		
	GP4	No Ct	39.94	No Ct	No Ct	No Ct	No Ct	N/A	N/A		
	GP5	No Ct	No Ct	No Ct	No Ct	No Ct	No Ct	N/A	N/A		
	GP6	39.81	No Ct	No Ct	No Ct	No Ct	No Ct	N/A	N/A		
4	GP1	No Ct	No Ct	No Ct	No Ct	No Ct	No Ct	No Ct	No Ct	26.67	No Ct
	GP2	No Ct	No Ct	No Ct	No Ct	No Ct	No Ct	No Ct	No Ct		
	GP3	No Ct	No Ct	No Ct	No Ct	No Ct	No Ct	No Ct	No Ct		
	GP4	No Ct	No Ct	No Ct	38.20	No Ct	39.95	N/A	N/A		
	GP5	No Ct	No Ct	No Ct	No Ct	No Ct	No Ct	N/A	N/A		
	GP6	No Ct	No Ct	No Ct	No Ct	No Ct	No Ct	N/A	N/A		
7	GP1	No Ct	No Ct	No Ct	No Ct	No Ct	No Ct	No Ct	No Ct	26.67	No Ct
	GP2	No Ct	No Ct	No Ct	No Ct	No Ct	No Ct	No Ct	No Ct		
	GP3	No Ct	No Ct	No Ct	No Ct	No Ct	No Ct	No Ct	39.95		
	GP4	No Ct	No Ct	38.03	No Ct	No Ct	No Ct	N/A	N/A		
	GP5	No Ct	No Ct	No Ct	No Ct	No Ct	No Ct	N/A	N/A		
	GP6	No Ct	39.69	No Ct	No Ct	No Ct	No Ct	N/A	N/A		
10	GP1	No Ct	No Ct	No Ct	No Ct	No Ct	No Ct	No Ct	No Ct	24.14	No Ct
	GP2	No Ct	No Ct	No Ct	No Ct	No Ct	No Ct	39.93	No Ct		
	GP3	No Ct	38.13	No Ct	39.29	No Ct	No Ct	No Ct	No Ct		
	GP4	No Ct	No Ct	No Ct	No Ct	No Ct	No Ct	N/A	N/A		
	GP5	No Ct	No Ct	No Ct	No Ct	No Ct	40	N/A	N/A		
	GP6	No Ct	No Ct	No Ct	No Ct	No Ct	No Ct	N/A	N/A		
14	GP1	No Ct	No Ct	No Ct	No Ct	No Ct	No Ct	No Ct	No Ct	24.81	No Ct
	GP2	No Ct	No Ct	No Ct	No Ct	No Ct	No Ct	No Ct	No Ct		
	GP3	No Ct	No Ct	No Ct	No Ct	39.02	No Ct	No Ct	No Ct		
	GP4	No Ct	39.93	38.45	No Ct	No Ct	No Ct	N/A	N/A		
	GP5	No Ct	No Ct	No Ct	No Ct	No Ct	No Ct	N/A	N/A		
	GP6	39.29	No Ct	No Ct	No Ct	No Ct	No Ct	N/A	N/A		

Note: Dpi, days post inoculation; GP, guinea pig; In, intraperitoneal injection; Sub, subcutaneous injection; N/A, not applicable.

**Table A2:** Detection of viral RNA (Ct values) in anal swabs from guinea pigs inoculated with AHSV/C using the real-time RT-PCR method.

Dpi	GP	10^7.5^ TCID_50_		10^6.5^ TCID_50_		10^5.5^ TCID_50_		Control Group		Positive Control	Negative Control
		In	Sub	In	Sub	In	Sub	In	Sub		
1	GP1	39.21	No Ct	No Ct	No Ct	No Ct	No Ct	38.15	No Ct	26.93	No Ct
	GP2	No Ct	No Ct	No Ct	No Ct	No Ct	No Ct	No Ct	No Ct		
	GP3	No Ct	39.43	No Ct	38.03	39.94	No Ct	No Ct	No Ct		
	GP4	No Ct	No Ct	39.65	No Ct	No Ct	No Ct	N/A	N/A		
	GP5	No Ct	No Ct	38.69	No Ct	No Ct	No Ct	N/A	N/A		
	GP6	39.15	No Ct	No Ct	No Ct	No Ct	No Ct	N/A	N/A		
4	GP1	No Ct	No Ct	No Ct	38.97	38.23	No Ct	No Ct	39.62	26.91	No Ct
	GP2	38.55	No Ct	38.27	No Ct	No Ct	No Ct	38.48	No Ct		
	GP3	No Ct	38.28	No Ct	No Ct	No Ct	No Ct	No Ct	No Ct		
	GP4	No Ct	No Ct	No Ct	38.73	No Ct	No Ct	N/A	N/A		
	GP5	No Ct	No Ct	38.37	No Ct	No Ct	38.77	N/A	N/A		
	GP6	39.91	No Ct	No Ct	39.78	No Ct	No Ct	N/A	N/A		
7	GP1	No Ct	No Ct	38.37	No Ct	No Ct	38.86	No Ct	38.46	26.73	No Ct
	GP2	No Ct	No Ct	No Ct	No Ct	No Ct	38.73	No Ct	No Ct		
	GP3	No Ct	38.24	No Ct	No Ct	No Ct	No Ct	No Ct	No Ct		
	GP4	No Ct	No Ct	No Ct	38.29	No Ct	No Ct	N/A	N/A		
	GP5	38.56	No Ct	No Ct	No Ct	No Ct	No Ct	N/A	N/A		
	GP6	No Ct	No Ct	No Ct	No Ct	No Ct	No Ct	N/A	N/A		
10	GP1	No Ct	No Ct	No Ct	38.73	No Ct	No Ct	No Ct	No Ct	26.91	No Ct
	GP2	No Ct	No Ct	No Ct	No Ct	No Ct	No Ct	39.60	No Ct		
	GP3	38.97	No Ct	No Ct	No Ct	38.33	No Ct	No Ct	No Ct		
	GP4	No Ct	39.76	38.85	No Ct	No Ct	No Ct	N/A	N/A		
	GP5	No Ct	No Ct	No Ct	No Ct	No Ct	No Ct	N/A	N/A		
	GP6	No Ct	No Ct	No Ct	No Ct	No Ct	No Ct	N/A	N/A		
14	GP1	No Ct	38.95	No Ct	No Ct	No Ct	No Ct	No Ct	No Ct	26.73	No Ct
	GP2	No Ct	No Ct	No Ct	38.35	No Ct	39.59	No Ct	No Ct		
	GP3	No Ct	No Ct	38.72	No Ct	No Ct	No Ct	No Ct	No Ct		
	GP4	No Ct	No Ct	No Ct	No Ct	No Ct	No Ct	N/A	N/A		
	GP5	No Ct	No Ct	No Ct	No Ct	No Ct	No Ct	N/A	N/A		
	GP6	No Ct	No Ct	No Ct	No Ct	No Ct	No Ct	N/A	N/A		

Note: Dpi, days post inoculation; GP, guinea pig; In, intraperitoneal injection; Sub, subcutaneous injection; N/A, not applicable.

**Table A3:** Serum neutralizing antibody titers (SN_50_) in guinea pigs inoculated with graded doses of AHSV/C (10^5.5^, 10^6.5^, 10^7.5^ TCID_50_) at different time points.

Dpi	10^7.5^ TCID_50_		10^6.5^ TCID_50_		10^5.5^ TCID_50_		Negative Control
	In	Sub	In	Sub	In	Sub	
7	-	-	-	-	-	-	-
14	4 ± 1	2 ± 0	2 ± 0	-	-	-	-
21	5 ± 1	2 ± 0	4 ± 2	2 ± 0	2 ± 0	-	-
28	7 ± 1	49 ± 4	5 ± 2	13 ± 4	4 ± 2	8 ± 2	-
35	8 ± 2	51 ± 5	5 ± 2	38 ± 3	6 ± 2	16 ± 3	-
42	23 ± 3	79 ± 8	16 ± 5	54 ± 5	13 ± 3	23 ± 3	-
49	84 ± 3	121 ± 9	22 ± 5	64 ± 4	20 ± 2	41 ± 4	-
56	91 ± 7	142 ± 5	28 ± 3	73 ± 8	22 ± 4	46 ± 3	-

Note: SN_50_ is the reciprocal of serum dilution for 50% virus neutralization, determined by the Reed–Muench method with three technical replicates per sample; data are presented as the mean ± standard deviation (SD). Dpi, days post inoculation; In, intraperitoneal injection; Sub, subcutaneous injection; -, not detected.

**Table A4:** Serum neutralizing antibody titers (SN_50_) in horses inoculated with AHSV/C (10^5.5^ TCID_50_) at different time points.

Dpi	H1	H2
7	-	-
14	-	-
21	8 ± 3	4 ± 1
28	40 ± 11	22 ± 5
35	56 ± 8	45 ± 7
Pre-inoculation negative control	-	-

Note: SN_50_ is the reciprocal of serum dilution for 50% virus neutralization, determined by the Reed–Muench method with three technical replicates per sample; data are presented as the mean ± standard deviation (SD). Dpi, days post inoculation; -, not detected; H1, horse 1; H2, horse 2.
