# Supplementary material for: Preliminary Assessment on Pathogenicity of an African Horse Sickness Virus Serotype 1 Strain in Guinea Pigs and Horses
Source: Microorganisms. 2026 Jul 16;14(7):1557. doi: 10.3390/microorganisms14071557 (PMC13414468; doi:10.3390/microorganisms14071557)
Supplement: Supplementary file 1 [file microorganisms-14-01557-s001.zip › Figure S2.pdf]

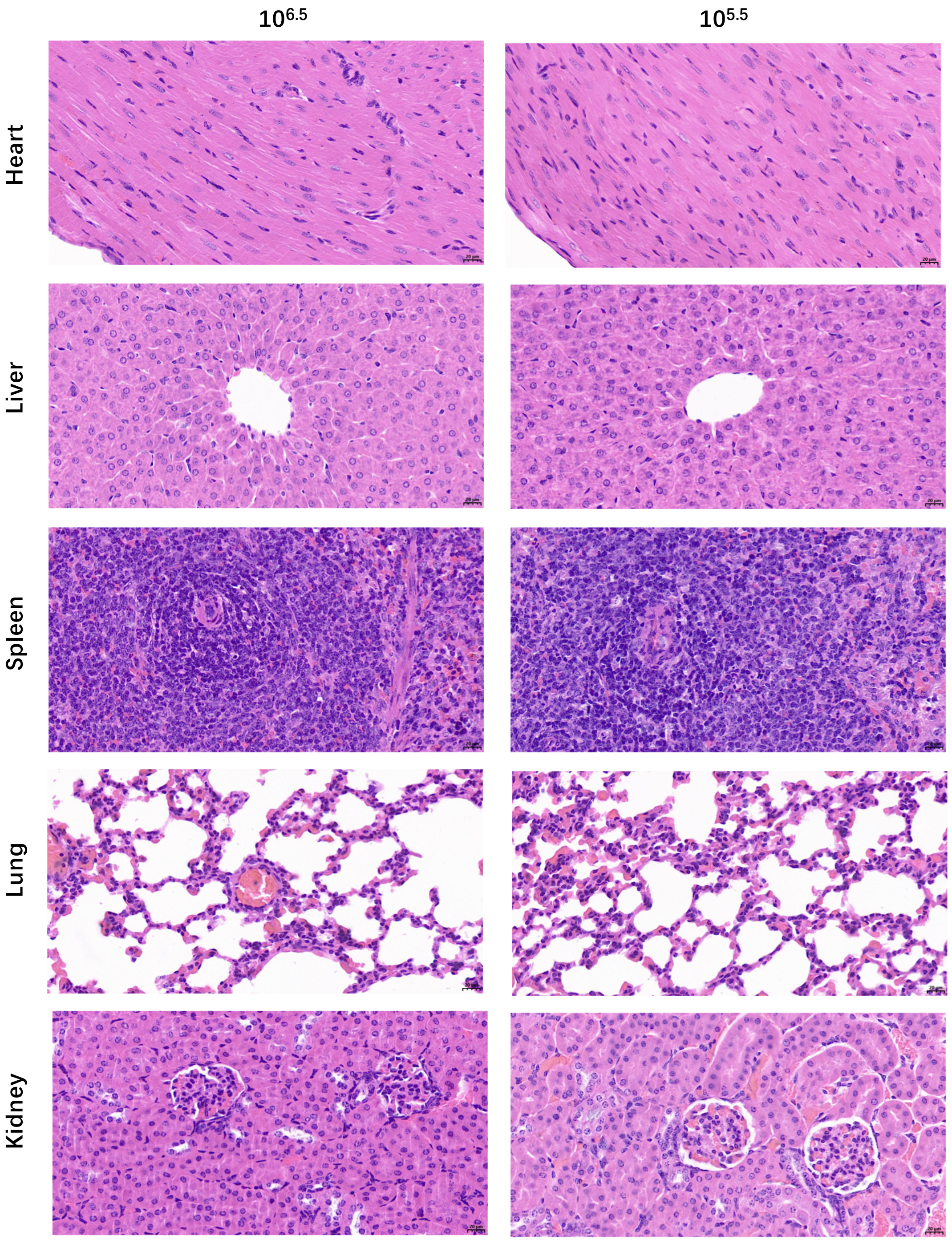
10^6.5
10^5.5
Heart
Liver
Spleen
Lung
Kidney

Figure S2. Microscopic features of the heart, liver, spleen, lung and kidney from guinea pigs subcutaneously injected with AHSV/C at a dose of $10^{6.5}$ and $10^{5.5}$ $TCID_{50}$/mL, as revealed by hematoxylin-eosin (HE) staining. No obvious histopathological lesions were observed in all the examined tissues. NC, negative control. Magnification: 400×.
